# Exploring Genomics and Microbial Ecology: Analysis of *Bidens pilosa* L. Genetic Structure and Soil Microbiome Diversity by RAD-Seq and Metabarcoding

**DOI:** 10.3390/plants13020221

**Published:** 2024-01-13

**Authors:** Wendy Lorena Reyes-Ardila, Paula Andrea Rugeles-Silva, Juan Diego Duque-Zapata, Glever Alexander Vélez-Martínez, Lina Tarazona Pulido, Karen Melissa Cardona Tobar, Sergio Alberto Díaz Gallo, Jaime Eduardo Muñoz Flórez, Lucia Ana Díaz-Ariza, Diana López-Alvarez

**Affiliations:** 1 Grupo de Investigación en Diversidad Biológica, Departamento de Ciencias Biológicas, Facultad de Ciencias Agropecuarias, Universidad Nacional de Colombia, Sede Palmira, Palmira 763533, Colombia; parugeless@unal.edu.co (P.A.R.-S.); jduquez@unal.edu.co (J.D.D.-Z.); glvelez@unal.edu.co (G.A.V.-M.);; 2 Grupo de Investigación en Agricultura Biológica, Departamento de Biología, Pontificia Universidad Javeriana, Sede Bogotá, Bogotá D.C. 110231, Colombia; sergio.diaz@javeriana.edu.co

**Keywords:** medicinal plants, genetic diversity, population structure, microbial communities

## Abstract

*Bidens pilosa* L., native to South America and commonly used for medicinal purposes, has been understudied at molecular and genomic levels and in its relationship with soil microorganisms. In this study, restriction site-associated DNA markers (RADseq) techniques were implemented to analyze genetic diversity and population structure, and metabarcoding to examine microbial composition in soils from Palmira, Sibundoy, and Bogotá, Colombia. A total of 2,984,123 loci and 3485 single nucleotide polymorphisms (SNPs) were identified, revealing a genetic variation of 12% between populations and 88% within individuals, and distributing the population into three main genetic groups, F_ST_ = 0.115 (*p* < 0.001) and F_IT_ = 0.013 (*p* > 0.05). In the soil analysis, significant correlations were found between effective cation exchange capacity (ECEC) and apparent density, soil texture, and levels of Mg and Fe, as well as negative correlations between ECEC and Mg, and Mg, Fe, and Ca. Proteobacteria and Ascomycota emerged as the predominant bacterial and fungal phyla, respectively. Analyses of alpha, beta, and multifactorial diversity highlight the influence of ecological and environmental factors on these microbial communities, revealing specific patterns of clustering and association between bacteria and fungi in the studied locations.

## 1. Introduction

*Bidens pilosa* L. (1753) belongs to the Asteraceae family and comprises 284 species (http://www.worldfloraonline.org, accessed on 2 October 2023). It is considered native to South America [1,2,3]. Its name comes from the Latin ‘bis’, signifying double or two, and ‘dens’, meaning tooth, while ‘pilosa’ denotes its soft-haired appearance. The plant features opposite, petiolate, pinnate leaves, characterized by 3–5 ovate leaflets with distinct dentate margins and slight hairiness. Recognizable by its elongated achene, bud-shaped with recurved or hook-shaped bristles, ensuring its dissemination. Its stems exhibit parallel lines or soft ridges, often displaying green hues with brown stripes. The inflorescence is a head with yellow centers and white ray petals, and the achenes are blackish, narrow, channeled, and with sparse to smooth bristles. The seeds are typically dark brown or black, slender, reaching a length of 1 cm, and cluster at the stem’s terminus [4]. As a perennial herb, *B. pilosa* boasts a widespread distribution across tropical and subtropical regions, thriving notably in countries like Colombia, Brazil, Peru, Uganda, Kenya, China, Australia, and Hawaii. It is considered an invasive plant that causes significant loss in agriculture, livestock, biodiversity, and forestry [5]. However, this species find utility in various communities for therapeutic and dietary purposes. In the Amazon, Uganda, and Africa, it serves as a food source, while in Kenya, the Himalayas, Australia, and Hawaii, it is harnessed for its therapeutic properties [4,6].

Currently, *B. pilosa* is utilized in traditional medicine to treat various ailments including asthma, pharyngitis, diabetes, gastritis, infectious diseases, and even cancer [7]. Extensive research has shed light on its medicinal potential, identifying 201 phytochemical compounds with therapeutic applications. Among these, polyynes, flavonoids, phenylpropanoids, fatty acids, polyacetylene compounds, and phenols stand out as the most important [1,7,8]. Notably, *B. pilosa* exhibits antitrypanosomal properties, particularly through the identification of butyl and propyl compounds of tryptophan. These compounds have been found to effectively inhibit the cell cycle of *Trypanosoma brucei* in Africa [9]. Despite being considered a weed in the main crops, various studies have been conducted on the microfauna associated with its rhizosphere. This attention has paved the way for its utilization in phytoremediation processes for soils contaminated by cadmium, owing to its remarkable capacity for metal adsorption facilitated by the presence of *Lysobacter* [10].

Although the impact of *B. pilosa* on soil ecosystems is significant, further research is essential for a comprehensive understanding [1]. This species modulates microbial activity, notably attenuating specific enzymatic activity, such as invertase, in full light environments, and alters nutrient dynamics by increasing the availability of nitrogen and potassium in the soil [11,12]. The carbon cycle can modify the transformation of organic matter into carbon and oxygen in the rhizosphere, thereby altering the decomposition of organic matter by microorganisms [11]. This change in microbial activity has significant implications for soil dynamics [13]. Additionally, *B. pilosa* influences the nitrogen cycle, affecting the transformation of atmospheric nitrogen into ammonia and its subsequent fixation into organic nitrogen compounds, which alters the availability of nitrogen in the soil [11,14].

Recent studies have examined the role of *B. pilosa* as a phytoremediator, with an emphasis on the carbon and nitrogen cycles in the rhizosphere. The application of *Enterobacter* sp. inoculants in the rhizosphere has revealed a substantial effect on these biogeochemical cycles [15]. Subsequent research highlights the influence of *B. pilosa* on the metabolic functions of the edaphic microbiota, underscoring its relevance in both medicinal applications and ecological research [5,16].

Observations of *B. pilosa*’s interactions prompt an exploration of how medicinal plants and soil microorganisms mutually benefit each other [17]. These plants not only have therapeutic value but also establish important symbiotic relationships with microorganisms, affecting soil health and plant growth [18]. The influence of these plants on their environment is partly due to the bioactive compounds they secrete, including secondary metabolites with therapeutic properties [19]. These aspects are key to understanding their role in the ecosystem and their potential medicinal use.

Therefore, the purpose of this study was to investigate and characterize the genetic diversity and population structure of *B. pilosa* an endemic plant of South America recognized for its medicinal uses, in three municipalities of Colombia: Palmira, Sibundoy, and Bogotá. For this, DNA sequencing associated with restriction sites (RADseq) was employed to identify single nucleotide polymorphisms (SNPs) and discover patterns of genetic variability at both inter and intrapopulation levels. Complementarily, metabarcoding along with physicochemical analysis was used to examine the microbial composition and assess the soil health in these municipalities. This multidimensional approach facilitated the correlation of genetic variability with ecological and environmental factors and the soil microbial composition, thus contributing to the understanding of how *B. pilosa* interacts with its environment and the potential impact of the associated microbiome on its medicinal properties [20].

## 2. Results

### 2.1. Genomic Data and SNP Calling

A total of 47,858,410 raw reads were obtained from 16 individuals of *B. pilosa*, with a GC content of 51.44%, the average reads per individual were 14,413,476 +/− 123,292 and all data had a Phred quality of Q30 > 90%. After the process radtags was performed, the same number of reads was maintained. Upon completing the pipeline in stacks, a catalog with 3,711,696 loci were obtained (Table S1). Eventually, 2,984,123 loci were genotyped with an effective coverage for sample of 9.2× (±2.7×). From these RAD tags, a total of 518,219 variants sites, 360,294 polymorphic sites, and 115,731 private alleles were obtained for three evaluated populations (Table 1). Subsequently, after applying a filter using tassel it was observed that the loci present in at least 70% of the sites were retained. Consequently, samples cor_2, 6, 7, and 8, Sib_2, and pal_1 were excluded for containing many missing data.

### 2.2. Genetic Diversity Analysis

The diversity analysis within natural populations (Table 2), indicated that the Palmira population samples exhibited the highest number of polymorphic loci and SNPs. Nevertheless, it was observed that all three populations showed similar nucleotide composition values, and in all three, the number of transitions is higher, suggesting that their evolution has been stable, with no apparent genetic drift processes. Furthermore, the samples from the Bogotá population showed a higher Ho value. This observation may be linked to the specific reproductive characteristics within the Juan N. Corpas germplasm bank.

### 2.3. Population Structure

**Figure 1:**
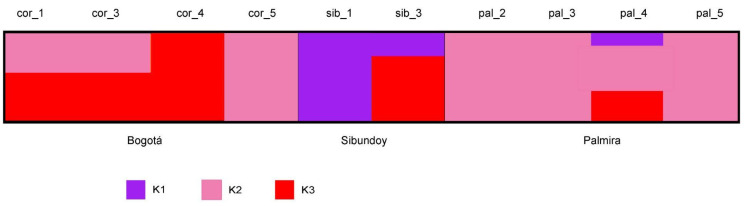
Analysis of population structure of 10 individuals based on estimation for K = 3 on 3485 polymorphic sites using ADMIXTURE.

**Figure 2:**
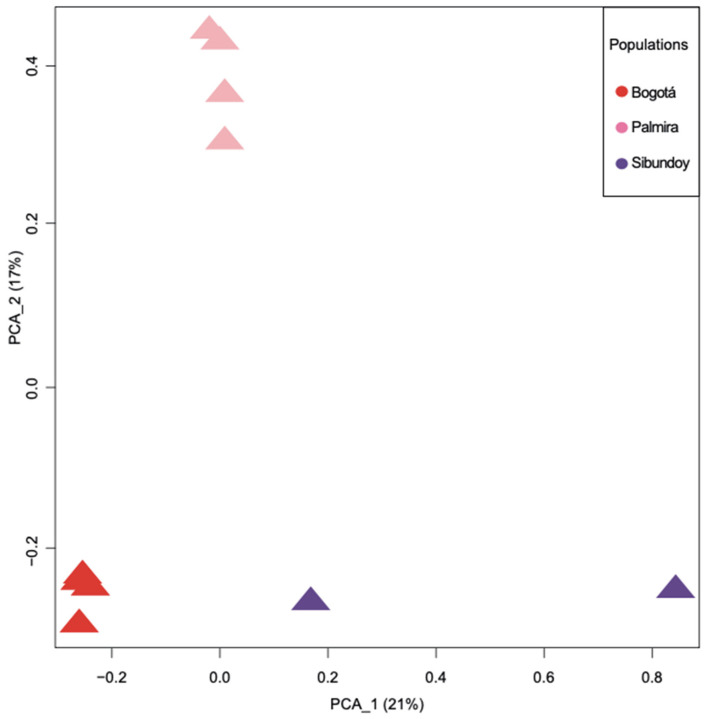
Principal Component Analysis for the three populations of *B. pilosa* based on 3485 polymorphic sites.

The ADMIXTURE analysis revealed the value at three delta (Δ) 3ΔK indicates that the optimal grouping for the studied populations. This grouping is clearly evident in the population structure diagram (Figure 1 and Figure S1). Notably, samples from Bogotá population, specifically those from the Juan N Corpas germplasm bank (cor_1, cor_3, cor_4, and cor_5), exhibit shared genetic information from both the K2 and K3 genetic groups (Table S2). In contrast, the sib_1 sample from the Sibundoy population exclusively contains genetic information from the K3 group, while the sib_3 sample contains information from both K1 (27%) and K3 (73%) groups. Furthermore, the samples from the Palmira population solely contain genetic information from the K2 group, except for the pal_4 sample, which exhibits information from all three genetic groups (K1 = 15%, K2 = 51%, K3 = 35%). This pattern is similarly reflected in the principal component analysis (Figure 2 and Figure S2), where the first component explains 21% of the variation, separating the samples from the Sibundoy and Palmira populations from those collected in Bogotá. Additionally, the second component explains 17% of the overall variance, specifically clusters samples associated with the Palmira population, separating them from all the others.

**Figure 3:**
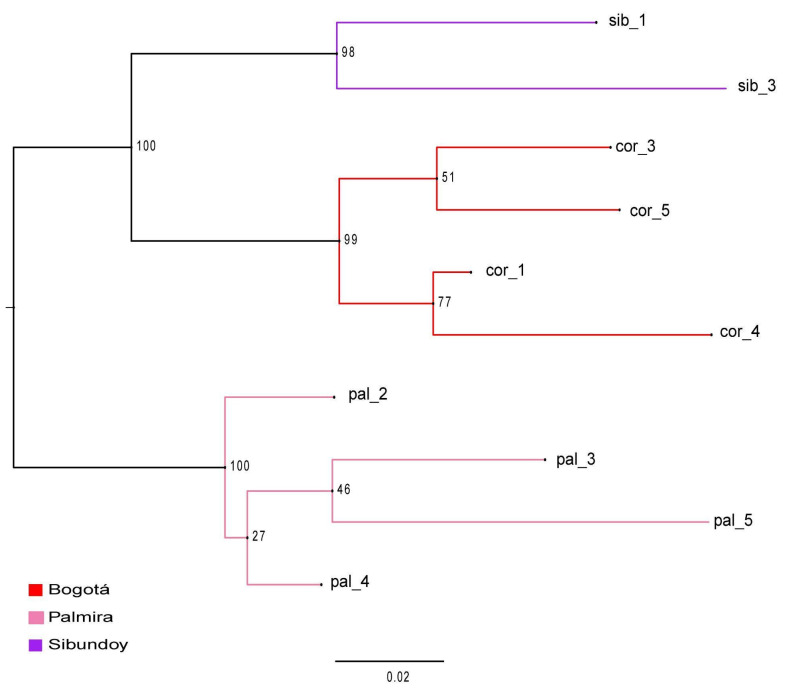
Phylogenetic tree estimated from Maximum Likelihood for three populations *B. pilosa*. In the nodes, bootstrap percentages.

The phylogenetic tree was constructed using the maximum likelihood model, revealing the formation of two clades, each supported by a robust Bootstrap value of 100% (Figure 3). Within the first clade, a further bifurcation revealed two subclades: one subclade grouped samples from the Sibundoy population, while the other contained samples from the Bogotá population. Furthermore, consistent with the PCA observations, the samples from Palmira constituted a distinct and separate cluster compared to the remaining samples.

The analysis of molecular variance effectively partitioned the overall genetic variance among the natural populations, attributing 12% (Table 3) of the variance with an F_ST_ value of 0.115 (*p* < 0.001). Moreover, the Nm value observed was 1.92, while the within-population diversity value, F_IT_, stood at 0.013 (*p* > 0.05). Regarding genetic differentiation, the pairwise values among populations ranged from 0.007 to 0.087 for the 1,023 loci utilized for distance computation. However, none of the population pairs exhibited significant differentiation (Table 4).

### 2.4. Physicochemical Analysis of Soils

**Figure 4:**
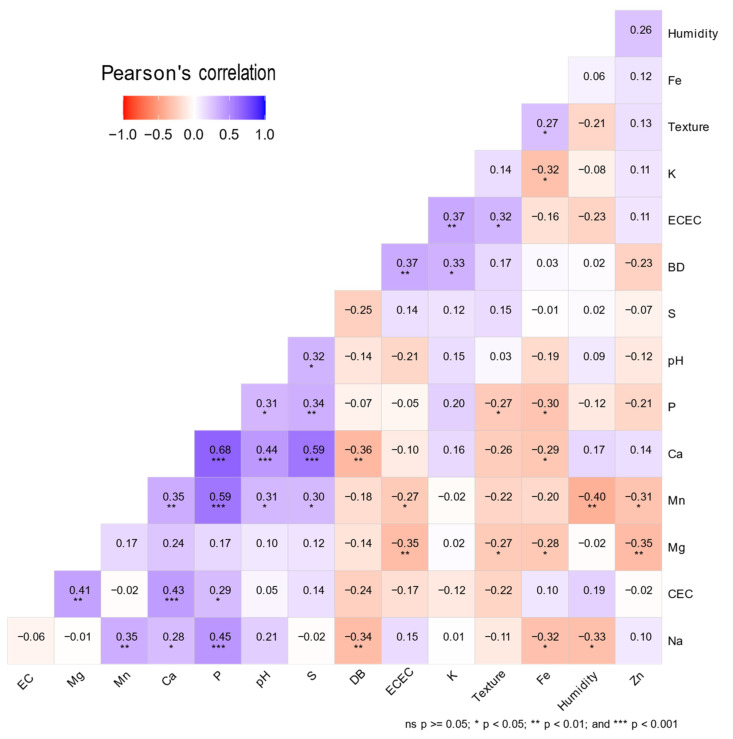
Heatmap of Pearson Correlation Coefficients for physicochemical variables of soil samples collected at sampling points in the populations of Bogotá, Sibundoy, and Palmira.

An analysis of the soil physicochemical properties was conducted employing Pearson correlation (Figure 4), complemented by significance tests to assess the strength of the observed relationships. The Effective Cation Exchange Capacity (ECEC) demonstrated moderate positive correlations with bulk density (BD) (r = 0.367, *p* < 0.05) and soil texture (r = 0.316, *p* < 0.05), suggesting that variations in these properties may be associated with changes in ECEC.

Soil texture revealed both negative and positive correlations with different elements. Specifically, correlations were observed with Magnesium (Mg) (r = −0.272, *p* = 0.043) and Iron (Fe) (r = 0.273, *p* = 0.042), respectively, indicating that changes in the proportion of clay, silt, and sand could influence the availability and retention of these nutrient elements in the soil.

On the other hand, a moderate positive correlation was identified between Calcium (Ca) and Sodium (Na) (r = 0.281, *p* = 0.036), suggesting that these two cations may share conditions influenced by agricultural practices or specific geological characteristics of the sampling location.

Additionally, a moderate negative correlation was found between ECEC and Mg levels (r = −0.349, *p* < 0.05), suggesting that, in the studied soils, an increase in ECEC is associated with a decrease in Mg concentration and vice versa. Furthermore, Mg also exhibited a negative correlation with Fe (r = −0.283, *p* = 0.035), potentially indicating that as Mg levels increase, Fe levels tend to decrease. Similarly, a moderate negative correlation was observed between Fe and Ca (r = −0.290, *p* = 0.030).

### 2.5. Microbiome Composition Analysis

Initially, a total of 661,427 bacteria readings were obtained, with 202,895 readings classified into 6261 ASVs retained after filtering and normalization processes (Figure S3A and Table S3). For fungi, 767,252 total reads were obtained, with 101,564 reads retained after preprocessing, resulting in the identification of 2019 ASVs (Figure S3E and Table S3).

At the phylum level, 100% assignment was achieved (Figure S3B), identifying 37 phyla. Among these, twenty-nine were shared across all populations, and one was exclusive to Sibundoy. At the class level (Figure S3C), a 99% assignment identified 92 classes, with 75 of them shared among all populations. Bogotá had one exclusive class, while Sibundoy and Palmira had two and three exclusive classes, respectively. Regarding genera (Figure S3D), a 43% assignment was achieved, with 327 identified. Of these, 41.3% were shared among all populations. Bogotá, Sibundoy, and Palmira had 10.7%, 6,1%, and 18.5% exclusive genera, respectively. As for fungi, 10 phyla were identified (Figure S3F), with Ascomycota and Basidiomycota representing 88% of the total relative abundance. Ascomycota was the most abundant at 58%. Among the three populations, Bogotá showed the highest relative abundance (64%), followed by Palmira and Sibundoy. Most phyla were shared among the populations, except Olpidiomycota, found solely in Bogotá despite its very low abundance (<1%).

**Figure 5:**
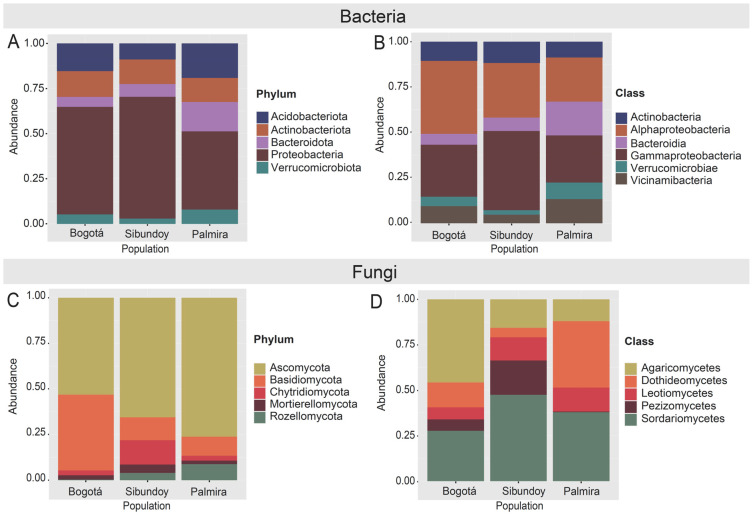
Relative abundance composition graphs. For bacteria: (**A**) Phylum, (**B**) Class. For fungi: (**C**) Phylum, (**D**) Class in soil samples of *B. pilosa*.

The analysis of bacterial composition highlighted Proteobacteria as the most abundant phylum across all sampling points, albeit with varying relative abundances. Palmira displayed a relatively even distribution among different bacterial groups (Figure 5A and Table S3), with Proteobacteria, Acidobacteriota, and Bacteroidota constituting 43.35%, 19.13%, and 16.22%, respectively.

In contrast, Sibundoy exhibited dominance by Proteobacteria, constituting over 67.54% of the bacterial population, while other groups were less represented, with Verrucomicrobiota being the most abundant at just 2.90%. Bogotá showcased significant representation of Acidobacteriota and Actinobacteriota, with relative abundances of 15.36% and 14.28%, respectively, alongside a notable presence of Proteobacteria.

At the class level (Figure 5B and Table S3), Gammaproteobacteria and Alphaproteobacteria were the most abundant classes across all sampling zones, accounting for 24% and 23%, respectively. Notable differences were observed in the abundance of Gammaproteobacteria among the sampling areas. Sibundoy exhibited the highest proportion at 43.6%, surpassing Bogotá (28.6%) and Palmira (26.0%). On the other hand, Alphaproteobacteria was more prevalent in Bogotá, accounting for 40.2% of the bacteria, compared to 30.1% in Sibundoy and 24.4% in Palmira.

Regarding Bacteroidia, they showed a clear preference for the soils of Palmira with an abundance of 18.6%, significantly higher than 6.0% in Bogotá and 7.4% in Sibundoy. In Bogotá, the percentages of Vicinamibacteria and Verrucomicrobiae were 9.4% and 5.2%, respectively. These groups were less prevalent in Sibundoy (4.7% and 2.5%, respectively) and more abundant in Palmira (13.3% and 9.1%), suggesting a greater affinity for the conditions of the latter municipality. Actinobacteria showed a more balanced distribution, with percentages of 10.5% in Bogotá, 11.7% in Sibundoy, and 8.6% in Palmira.

Finally, at the genus level for bacteria, it was found that in Bogotá, *Acinetobacter* (24%), *Sphingomonas* (18.9%), and *Pseudomonas* (23.4%) were the most abundant taxa. In Sibundoy, *Acinetobacter* was also predominant, with a relative abundance of 45.2%, followed by *Pseudomonas* with 31.8%. In Palmira, *Sphingomonas* had the highest abundance at 49%, being the most prevalent taxon in this area. The DESeq2 analysis (Figure S4A,B) for bacteria revealed differences in 23 genera between the Palmira and Bogotá populations, which are part of the most abundant phyla, except for *Nitrospira*, which belongs to Nitrospirota.

Concerning fungi (Figure 5), Sibundoy exhibited an increased abundance in the phyla Chytridiomycota and Mortierellomycota (Figure 5C and Table S3), while Rozellomycota shows a lower abundance in Palmira. At the class level, 29 classes were identified. The top five most abundant classes—Agaricomycetes (30%), Sordariomycetes (28%), Dothideomycetes (14%), Leotiomycetes (7%), and Pezizomycetes (6%)—accounted for 86% of the total relative abundance (Figure 5D and Table S3). Specifically, Agaricomycetes predominated in the Bogotá samples, representing 41% of their total abundance. Conversely, Dothideomycetes exhibits its highest prevalence in Palmira, constituting 40% of this class’s total abundance. The DESeq analysis at the genus level (Figure S4) identified 164 genera. Notably, three genera—*Pseudopithomyces*, *Ascobolus*, *Minimedusa*—showed differences in abundance between the Palmira and Bogotá populations. Moreover, *Ascobolus* and *Minimedusa* displayed differences between Sibundoy and Bogotá, being more abundant in the latter locality (Figure S4).

### 2.6. Alpha and Beta Diversity of Bacteria and Fungi

**Figure 6:**
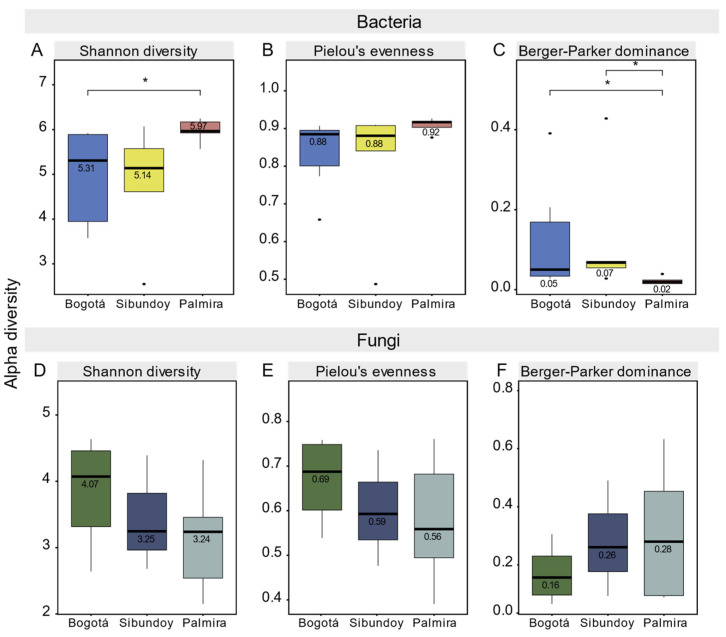
Alpha diversity plots for bacteria and fungi associated with *B. pilosa* Asterisks denote significant differences between populations: * *p* < 0.05.

This study analyzes the alpha diversity of bacteria and fungi in the sampled populations, as illustrated in Figure 6 and detailed in Table S5. In Bogotá, the Shannon index for bacteria is median $\tilde{x}\;$ 5.31, indicating high diversity (Figure 6A). Pielou’s Evenness, with a median of $\tilde{x}\;$ 0.88 (Figure 6B), and Berger–Parker Dominance, with $\tilde{x}$ 0.05 (Figure 6C), suggest an equitable species distribution and low individual dominance, respectively. In contrast, fungi in Bogotá show a Shannon index of $\tilde{x}\;$ 4.07 (Figure 6D), with Pielou’s Evenness and Berger–Parker Dominance values of $\tilde{x}$ 0.69 (Figure 6E) and $\tilde{x\;}$ 0.16 (Figure 6F), respectively, reflecting a distribution like that observed in bacteria.

In Palmira, bacteria record the highest Shannon index, $\tilde{x}\;$ 5.97 (Figure 6A), with the highest Pielou’s Evenness, $\tilde{x}$ 0.92 (Figure 6B), and a notably low Berger–Parker Dominance, $\tilde{x}\;$ 0.02 (Figure 6C). In comparison, fungi in Palmira show a Shannon index of $\tilde{x}\;$ 3.24 (Figure 6D), with a Pielou’s Evenness of $\tilde{x}\;$ 0.56 (Figure 6E) and a Berger–Parker Dominance of $\tilde{x}\;$ 0.28 (Figure 6F), indicating high biological diversity.

In Sibundoy, it was observed that bacteria exhibit a Shannon index of $\tilde{x}$ 5.14 (Figure 6A), indicating specific diversity. This diversity is accompanied by a Pielou’s Evenness of $\tilde{x}$ 0.88 (Figure 6B), a figure that matches exactly with the one recorded in Bogotá, suggesting similarities in the equitable distribution of species between both sites. Additionally, the Berger–Parker Dominance of $\tilde{x}$ 0.07 (Figure 6C) is comparable to that observed in Bogotá, indicating a similar trend in terms of predominant species.

Regarding the fungi in Sibundoy, a Shannon index of $\tilde{x}$ 3.25 (Figure 6D) is reported. The Pielou’s Evenness and Berger–Parker Dominance values are $\tilde{x}$ 0.56 (Figure 6E) and $\tilde{x}$ 0.26 (Figure 6F), respectively. These results reflect a parallel trend to the one already mentioned for Bogotá, which may indicate similar patterns in the distribution and dominance of fungal species in both populations.

**Figure 7:**
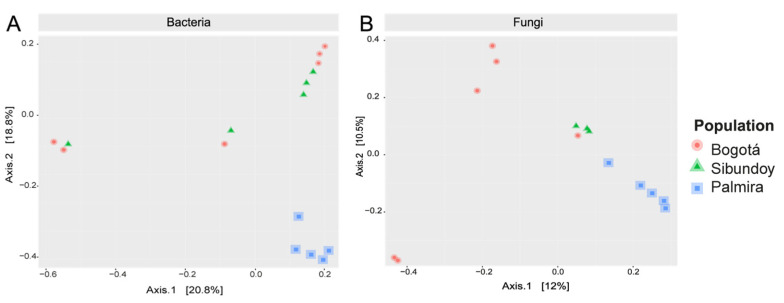
Principal coordinates analysis plots for bacterial and fungal communities based on Bray–Curtis distances.

As for beta diversity, the PCoA plot for bacteria (Figure 7A) explained 20.8% of the variance on the first axis and showed the separation of Palmira and six samples from Bogotá and Sibundoy, distinct from five samples from these latter populations. The second axis, accounting for 18.8% of the variance, delineated a grouping of the six Bogotá and Sibundoy samples from the remaining samples. On the other hand, the first axis of the PCoA plot for fungi (Figure 7B) explained 12% of the variance and separated the Palmira and Sibundoy samples from most of the Bogotá samples. The second axis, with 10.5% of the variance, separated four samples from Bogotá and all Sibundoy from Palmira, and two from Bogotá.

**Figure 8:**
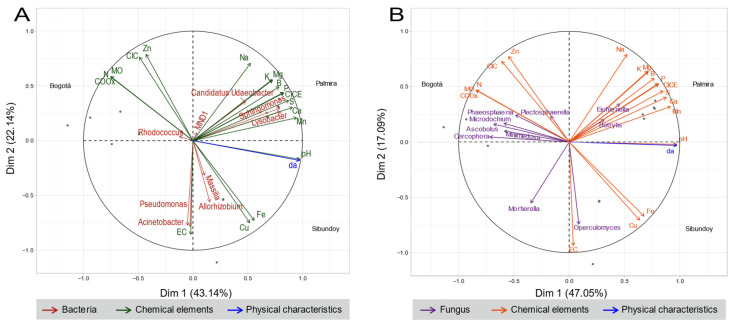
Multifactorial analysis of the physicochemical properties of soil and their relationship with the most abundant genera of (**A**) bacteria and (**B**) fungi. Red arrows represent genus of bacteria, while purple arrows represents genus of fungi.

A Multifactorial Analysis (MFA) was performed, as shown in Figure 8, demonstrating a contribution of 65.28% and 64.14% for both bacteria and fungi, respectively, across their two dimensions. This reflects a correlation between microorganisms and the analyzed physicochemical variables. In the first dimension, for both bacteria and fungi, a significant contribution of the abiotic parameter bulk density is evident, with Euclidean distances of 40.2 and 37.5, respectively.

For bacteria (Figure 8A), in dimension 1, the microorganisms *Sphingomonas* and *Lysobacter* are associated with the elements pH and Mn. In dimension 2, *Acinetobacter*, *Pseudomonas*, and *Allorhizobium* show a stronger relationship with EC, Zn, CEC, Cu, Fe, Na, Mn, and pH values. The contributions of SOM, N, and COOx were relatively equal across both dimensions, suggesting an indirect influence on microorganism abundance.

Contrastingly, for fungi (Figure 8B), dimension 1 revealed relationships between *Cercophora*, *Ascobolus*, *Microdochium*, and *Minimedusa* with the elements pH, SOM, N, COOx, S, and Ca. Contributions of p and CEC were equivalent for both dimensions. In dimension 2, *Operculomyces*, *Mortierella*, and *Eichleriella* exhibited a more substantial contribution and were associated with EC, Na, Zn, CEC, Fe, Mg, K, and B.

## 3. Discussion

This study expands the understanding of the genetic diversity and population structure of *B. pilosa*, a species identified as a weed [21], with various food uses and in traditional medicine [2,22]. Although its role in phytoremediation, especially in the absorption of cadmium [10,22], and its therapeutic potential are well documented [7], detailed genomic research is scarce. Our work endeavors to fill this gap by exploring the genetics of *B. pilosa* and scrutinizing the interactions between soil microorganisms, the plant, and its environment. The analysis conducted in the municipalities of Bogotá, Sibundoy, and Palmira in Colombia revealed distinct patterns in bacterial and fungal distribution and composition, shedding light on microbial diversity.

The genetic diversity of *B. pilosa* was studied using Bayesian analysis, identifying three distinct genetic groups (K = 3) associated with specific sampling zones. A moderate level of genetic differentiation was observed, with an F_ST_ value of 0.115 (*p* < 0.001), which is complemented by an F_IT_ of 0.013 (*p* > 0.05), indicating relatively low genetic differentiation among populations [23,24]. These findings are consistent with previous studies on *Bidens* species [25], where they found broad intraspecific genetic variability, evidenced through ISSR polymorphism analysis. The observed genetic diversity in *B. pilosa* may suggest a relationship with its seed dispersal methods. Specifically, the production of achenes with hooks that adhere to animal fur [26] facilitates localized dispersal and may contribute to the observed genetic variability, limiting genetic exchange over broader geographical distances.

Consequently, the research on the chloroplast genomes of endemic chinese plants [27] related to *B. pilosa* reflects a phylogenetic grouping consistent with their geographic origins. This grouping suggests selective adaptations, likely influenced by the unique environmental conditions of each location [28]. Furthermore, significant variations in plastomes among individuals from different habitats indicate considerable genetic diversity, underscoring the adaptability of *B. pilosa* to various environmental conditions [27].

Our findings align with this research, as the three studied populations clustered in the phylogenetic tree according to their geographic origins. This grouping is likely associated with the distinct environmental characteristics of each location. Supported by other studies [29,30] that analyzed complete plastomes of *B. pilosa* from Beijing using the NOVO-plastia technique, our results indicate significant plastome variation among different *B. pilosa* plants. These results suggest considerable genetic diversity among individuals from varied habitats.

Notably, soil, as a complex ecosystem, differs distinctly among geographical locations and profoundly impacts and is impacted by the plant it harbors, as observed in other studies on *B. pilosa* [31]. The physical, chemical, and biological components of the soil, such as its texture, nutritional composition, and microbial communities, play crucial roles in the health of the plant and its ability to adapt to different environments [32]. Understanding the dynamics of *B. pilosa* with its edaphic environment is not only fundamental for understanding its ecology and evolution but also for practical applications in its management.

Therefore, this study also delved into investigating soil microorganisms, with an emphasis on the interactions between microbial communities, *B. pilosa*, and their environment. Specific patterns in the distribution and composition of bacteria and fungi were identified. The detection of variability in microbial composition provides an initial and detailed perspective on the microbial diversity present in each municipality.

Proteobacteria emerges as the predominant phylum in all populations, consistent with previous studies [33,34]. Palmira shows a high diversity and evenness of bacteria, reflected in the Principal Coordinates Analysis (PCoA), suggesting a more heterogeneous distribution and lower dominance of specific species. This contrasts Bogotá and Sibundoy, while displaying less bacterial diversity, showcase unique characteristics with significant representation of Acidobacteriota and Actinobacteriota. Concerning fungi, Ascomycota and Basidiomycota prevail, playing vital ecological roles. Noteworthy adaptations are observed in Bogotá (Agaricomycetes) and Palmira (Dothideomycetes), reflecting specific environmental adaptations [35]. The PCoA analysis supports these findings, displaying distinct groupings of fungal communities that imply localized adaptation and differentiation among the study areas [36], potentially in response to distinct genetic groups of *B. pilosa*.

The analysis of Pielou’s evenness and Berger–Parker Dominance unveils relatively uniform species distribution across populations, with notable variations between bacteria and fungi. Specifically, fluctuations in Berger–Parker Dominance in Sibundoy might mirror environmental differences, consistent with observed microbial community differentiations in the PCoA analysis. These analyses highlight Palmira’s distinct bacterial diversity and distribution, corroborated by high bacterial evenness and variability in Berger–Parker dominance. Conversely, while Bogotá and Sibundoy exhibit lower bacterial diversity overall, their unique patterns of Acidobacteriota and Actinobacteriota, along with fungal differentiation in PCoA, underscore their essential ecological roles. The prevalence of Ascomycota and Basidiomycota, documented in various ecosystems [27], is consistent with their predominance in Colombian soils across departments [37].

Similarly, the physicochemical properties of the soil, such as effective cation exchange capacity (ECEC), soil texture, and concentrations of elements like Mg, Fe, Ca, and Na, not only have direct implications on plant nutrition and health but also affect the composition and function of soil microorganisms [16]. Correlations found between ECEC, Mg, and Fe with soil texture and other nutritional elements suggest that changes in these soil properties can have direct or indirect effects on the genetic diversity of plants and the composition of the microbiome [38,39]. For instance, a higher ECEC could influence the availability of nutrients for plants and microorganisms, while soil texture affects water and nutrient retention, impacting the living conditions of both [40].

However, the Multifactorial Analysis (MFA) provided a deeper insight into the relationship between microorganisms and physicochemical variables. Apparent density was highlighted as an influential abiotic factor, along with parameters such as pH, metals (Zn, Cu, Fe, Mn), and nutrients (N, P, SOM, CEC) [32,33]. These relationships were specific to different bacterial and fungal genera, reflecting how environmental conditions can favor or inhibit certain microbial communities. In the case of bacteria, genera like *Sphingomonas* and *Lysobacter* from the Proteobacteria phylum were associated with pH and Mn, while *Acinetobacter* and *Pseudomonas* from the same phylum showed a stronger relationship with a wider range of physicochemical elements (EC, Zn, CEC, Cu, Fe, Na, Mn, pH).

In this context, a study in China on the invasion of *B. pilosa* indicates significant alterations in microbial composition and soil physicochemical properties, evidenced by increases in organic matter, total nitrogen, and nitrate, as well as rhizospheric enzymatic activity [5]. These changes suggest an alteration in the biogeochemical cycles and microbial structure, demonstrating the soil’s vulnerability to biotic influences. Complementarily, studies that incorporated inoculation with *Enterobacter* sp. FM-1 [15] and increased salinity [41], underline how abiotic factors can further alter microbial diversity and plant-soil interactions.

Finally, the genetic diversity of *B. pilosa*, reflected in the number of polymorphic loci, variant sites, and private alleles, suggests adaptability and evolution that are intrinsically linked to environmental conditions, including the physicochemical properties of the soil and microbial interactions. The differences observed between the populations of Bogotá, Sibundoy, and Palmira indicate that the plants may be adapting to specific local conditions, highlighting their high phenotypic plasticity [42]. This adaptability likely results from natural selection acting on genetic variants better suited to local soil conditions and prevalent microbial regimes. Similar observations in *Medicago truncatula*, where microbiome diversity and composition significantly correlate with soil origin and plant genetics [43]. Underscore the importance of considering genetic and environmental influences, including microbial communities, in studying plant adaptability and evolution. These findings in *B. pilosa* offer a comparative framework to comprehend species-specific responses to their environments, contributing to a deeper understanding of adaptive mechanisms.

## 4. Materials and Methods

### 4.1. Collection of Material

**Figure 9:**
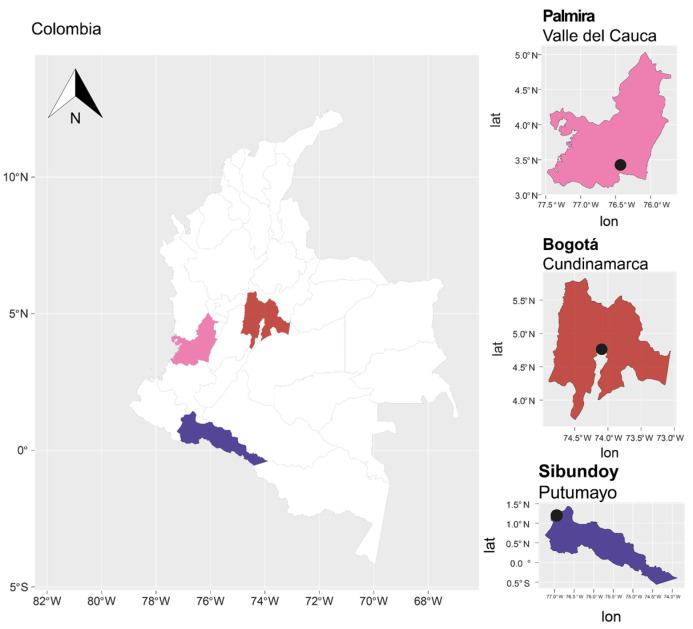
Location map of the sampling sites.

16 individuals of *B. pilosa* were selected from three locations in Colombia: five from the Experimental Center of the Universidad Nacional de Colombia (UNAL) in Palmira, Valle del Cauca; eight from the germplasm bank of the Fundación Universitaria Juan N. Corpas in Bogotá, Cundinamarca; and three from Sibundoy, Putumayo (Table S4 and Figure 9).

During sampling, between three and five young leaves were collected from each individual, ensuring they were free from signs of predation or disease. These samples were immediately stored in paper bags with silica gel to preserve their integrity by reducing moisture and preventing phytosanitary issues until further analysis in the laboratory.

Concurrently with leaf collection, approximately 1 kg of soil samples were extracted at a depth of 0 to 20 cm, removing the organic matter from the O horizon for physicochemical analysis. Additionally, 1 g of soil associated with the root of each individual was obtained to extract environmental DNA to identify the present microorganisms. These soil samples were placed in microcentrifuge tubes and stored at −80 °C until their subsequent analysis in the laboratory.

### 4.2. DNA Extraction and Sequencing

For the extraction of DNA from plant samples, the process began with the macerated of leaves using liquid nitrogen, followed by the purification of 40 mg of homogenized using the PROMEGA Wizard^®^ Genomic DNA Purification Kit, following the manufacturer’s protocol. Concurrently, microbial DNA was obtained from soil samples following the Doyle and Doyle protocol [44].

Subsequently, the quality of the DNA from both sample types, including purity and integrity, was assessed using the Qubit 4™ DNA Assays, Kit Qubit™ 1X dsDNA High Sensitivity (HS) Fluorometer (Invitrogen by Thermo Fisher Scientific, Waltham, MA, USA). Additionally, the Colibri™ spectrophotometer (Titertek Berthold, Neulingen, Germany) and agarose gel electrophoresis at 0.8% with TBE 0.5 provided further quality parameters, thus ensuring the suitability of the DNA.

The genome sequencing technique for *B. pilosa* was restriction site associated DNA markers (RADseq), using the type II restriction enzyme PstI. This process was conducted in the FLORAGENEX, Inc. laboratory (https://www.floragenex.com, Beaverton, OR, USA), following the protocol [45]. The amplified library was purified and size-selected using Agencourt AMPure XP beads from Beckman Coulter Life Sciences, and the size distribution was determined using an Agilent bioanalyzer with a high-sensitivity kit. This step provided an average fragment size of 580 to 602 base pairs, suitable for subsequent sequencing using the Illumina NovSeq 6000 at the Central Facility for Genomic and Cellular Characterization (GC3F) of the University of Oregon. The raw RAD sequence has been deposited in the National Center for Biotechnology Information (NCBI) Bioproject under the accession number PRJNA1025911, and the samples among SAMN39418942 a la SAMN39418957 number.

Soil metabarcoding was performed by the Argonne National Laboratory of the U.S. Department of Energy. This included the preparation of the library for the V4-V5 region of the 16S ribosomal gene for bacteria, following the methodology of Caporaso [46] and ITS region using the ITS1F/ITS2 primers. Sequencing was conducted using Illumina HiSeq2000 and MiSeq technologies. The results of the sample readings were deposited in the European Nucleotide Archive (ENA) under project number PRJEB67294 and access numbers ERS16459342 to ERS1645938.

### 4.3. Genetic Diversity and Population Structure of B. pilosa

Quality assessment was conducted using FastQC software v.0.12.1 [47]. We identified single nucleotide polymorphisms (SNPs) using the open-source software STACKS v.6.5 [48,49,50,51]. The process_radtags tool was used to demultiplexing the reads, retaining all sequences with a Phred quality score greater than 10 (default value) and discarding incorrectly identified (N), and creating consensus loci catalogs with the cstacks tool. The sequences were organized into a popmap by sample location. This facilitated the comparison of catalog samples with the sstacks tool. Additionally, using tsv2bam, data were organized by locus instead of by sample.

With the RAD data catalog, we used the gstacks tool to identify SNPs within the metapopulation. This was completed under the marukilow model with a predetermined Alpha threshold of 0.005, achieving individual genotyping [52]. Prior to this, the STACKS v.2.5 software’s population tool [50] was used to obtain the Variant Calling file (.vcf) containing the identified SNPs. A final quality filter was applied in Tassel [53], ensuring that the identified loci were present in at least 70% of the individuals.

The .vcf file was transformed for use in genetic group analysis using the open-source tool PLINK v2.0 [54]. Model-based clustering analysis was conducted through ADMIXTURE v.1.3.0 [55]. STRUCTURE SELECTOR [56] analyzed the group structure, employing the .Q and .P files along with a population map. We present the number of subgroups (K value) from 1 to 5 for clustering. The clustering results were cross-validated to determine the optimal number of groups based on the lowest cross-validation error rate, considering the analyzed natural populations and more than 10,000 bootstraps [57,58,59]. Principal Component Analysis (PCA) was performed in R software v4.3.1 [60] using the ggplot2 package [61]. Phylogenetic relationships were deduced using IQ-TREE v 2.2.2.6 [62] a software for maximum likelihood analysis of large phylogenetic data with a robust Bootstrap value of 1000.

For the genetic groups identified in ADMIXTURE v.1.3.0 [55], we conducted a diversity analysis. This included obtaining genetic statistics such as observed heterozygosity (Ho), expected heterozygosity (He), transitions and transversions numbers, nucleotide compositions, and diversity (π). We also calculated genetic differentiation (F_ST_) and the total inbreeding coefficient (F_IT_) was determined based on 10,000 permutations. Additionally, we evaluated the Nm parameter using the formula Nm  =  (1 − F_ST_)/(4F_ST_). A Molecular Variance Analysis (AMOVA) was conducted using Arlequin v 3.5.2.2 [63].

### 4.4. Physicochemical and Microbial Sequence Analysis in Soils

The physicochemical analysis of the soils was conducted at the AGRILAB laboratory in Bogotá, Colombia. Various variables were evaluated such as pH, effective cation exchange capacity (ECEC), electrical conductivity (EC), as well as the percentage of soil organic matter (SOM), percentage of oxidizable organic carbon (COOx), percentage of total nitrogen (N), and various elements like phosphorus (P) (mg/kg), sulfur (S) (mg/kg), calcium (Ca) (meq/100), magnesium (Mg) (meq/100), potassium (K) (meq/100), sodium (Na) (meq/100), boron (B) (mg/kg), iron (Fe) (mg/kg), copper (Cu) (mg/kg), manganese (Mn) (mg/kg), and zinc (Zn) (mg/kg). Physical variables, such as bulk density (BD) (g/cm^3^) and texture, were also considered. The obtained results were analyzed using a Pearson correlation test and visualized through a heatmap in R, version 4.2.2. Additionally, a Multifactorial Analysis (MFA) was applied to evaluate the relationships between the physicochemical variables and the sampling sites, using the FactoMineR package in R.

Subsequently, the bioinformatic analysis was carried out. The QIIME2 software version 2023.7 [64] was used for quality control, employing the DEMUX plugin. This included the removal of noise and chimeras using DADA2. Next, the taxonomic assignment of the samples was performed using the databases: SILVA [65] for bacteria and UNITE [66] for fungi. To normalize the data, bacterial and fungal sample reads were adjusted to 15,700 and 19,104, respectively. For the analysis of these, the R program version 4.2.3 and the packages qiime2R v. 0.99.6 and phyloseq [67] were used, which allowed loading the QIIME2 artifacts and generating Venn diagrams and additional analyses. Differential abundance analyses DESeq2 [68] were performed at the genus level for fungi and bacteria.

Finally, microbial diversity was assessed using alpha diversity indices, including Shannon diversity, Berger–Parker dominance, and Pielou’s evenness, using the microbiome package from Bioconductor. Beta diversity was examined through a Principal Coordinates Analysis (PCoA) based on Bray–Curtis distances. Complementarily, a multifactorial analysis was carried out to explore the correlation between the abundances of the microbiota and the physicochemical measurements of the soil.

## 5. Conclusions

The research conducted into *B. pilosa* revealed remarkable diversity aligned with geographical locations, showcasing variability within individuals and distinct clustering based on geographic sites. Employing a de novo assembly methodology facilitated a comprehensive assessment of genetic diversity and population structure within this species. The identification of SNPs holds significant promise for conducting in-depth analyses, especially in exploring associations between genetic diversity and the production of secondary metabolites with pharmacological potential. Our study unveiled substantial diversity among bacteria and fungi associated with *B. pilosa*, notably highlighting the pivotal ecological roles of Ascomycota and Basidiomycota in soil processes. The dominance of Proteobacteria in Bogotá and Sibundoy contrasts with Palmira’s notably rich bacterial diversity. Beta diversity patterns and correlations with soil physicochemical parameters underscore the intricate relationship between microorganisms and their habitat. Soil density and nutrient accessibility emerged as crucial determinants influencing microbial communities. A multifactorial analysis identified specific microbes associated with soil chemical parameters, indicating their influence on microbial composition. This suggests that soil management practices and geological features may significantly impact these complex dynamics. Finally, this study underscores the significance of *B. pilosa*’s genetic diversity, the potential implications of identified SNPs, and the intricate relationship between microorganisms and soil parameters. These findings have broad implications for agricultural and medical contexts, hinting at potential applications in enhancing agricultural practices and uncovering pharmacologically relevant compounds.

## Figures and Tables

**Table 1:** Population summary Stacks v 2.64.

Population	Variant	Polymorphic Sites	Private Alleles
Bogotá	181,987	121,310	45,318
Sibundoy	115,553	54,323	30,557
Palmira	220,679	184,661	39,856

**Table 2:** Genetic diversity indices of three populations of *B. pilosa* based on 3485 SNP markers.

Pop	Usable Loci	Ts	Tv	Polymorphic Locus	Na	SNPs	Nucleotide Composition %				π	Ho	He
							C	T	A	G			
Bogotá	2407	722	389	121,310	45,318	1111	25.33	25.14	25.13	24.40	0.361 +/− 0.236	0.674	0.574
Sibundoy	1837	632	336	54,323	30,557	968	25.94	25.22	24.77	24.07	0.422 +/− 0.276	0.551	0.561
Palmira	1597	655	391	184,661	39,856	1406	25.83	25.28	24.47	24.42	0.420 +/− 0.229	0.512	0.444

Pop = populations, Ts = number of transitions, Tv = number of transversions, SNPs = number of polymorphic sites; Na = Number of alleles; C = Cytosine; T = Timine; A = Adenine, G = Guanine; π = nucleotide diversity; Ho = observed heterozygosity; He = expected heterozygosity.

**Table 3:** Analyses of molecular variance (AMOVA) for three populations of *B. pilosa*.

Source of Variation	df.	Sum of Squares	Variance Components	Percentage of Variation	Fixation Indices
Among populations	2	1200.527	50.537	12	F_ST_ = 0.115 (*p* < 0.001)
Within individuals	7	4363.083	388.927	88	F_IT_ = 0.013 (*p* > 0.05)

**Table 4:** Pairwise F_ST_ values for three populations of *B. pilosa* F_ST_ values are shown above diagonal, and their *p* values are below diagonal.

	Bogotá	Sibundoy	Palmira
Bogotá	0	0.007	0.056
Sibundoy	0.329 ± 0.011	0	0.087
Palmira	0.067 ± 0.008	0.061 ± 0.008	0

## Data Availability

All data generated or analyzed during this study are included in this published article.

## Supplementary Materials

The following supporting information can be downloaded at: https://www.mdpi.com/article/10.3390/plants13020221/s1, Figure S1: CLUMPAK main pipeline population structure; Figure S2: PCA for 4 components; Figure S3: Venn diagrams for bacteria (A–D) and fungi (E–H) for the medicinal plant *B. pilosa*; Figure S4: Differential abundance plots at the genus level for bacteria (A,B) and fungi (C,D) from the *B. pilosa* plant; Table S1: Pipeline Stacks results; Table S2: K values for samples of three populations; Table S3: Total ASV and reads obtained per municipality and retained after normalization and processing steps; Table S4: Sampling site information; Table S5: Alpha Diversity.
