# Supplementary material for: Exploring Genomics and Microbial Ecology: Analysis of Bidens pilosa L. Genetic Structure and Soil Microbiome Diversity by RAD-Seq and Metabarcoding
Source: Plants (Basel). 2024 Jan 13;13(2):221. doi: 10.3390/plants13020221 (PMC10818919; doi:10.3390/plants13020221)
Supplement: Supplementary file 1 [file plants-13-00221-s001.zip › Figure S1. CLUMPAK main pipeline population structure.pdf]

CLUMPAK main pipeline - Job 1698372789 summary

Major modes for the uploaded data:

K=1

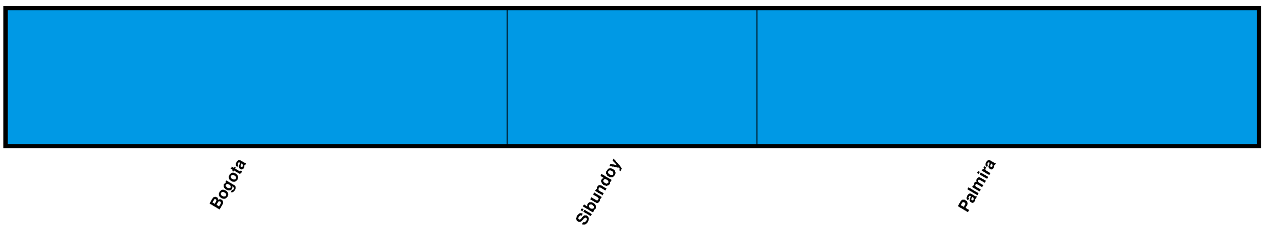

K=2

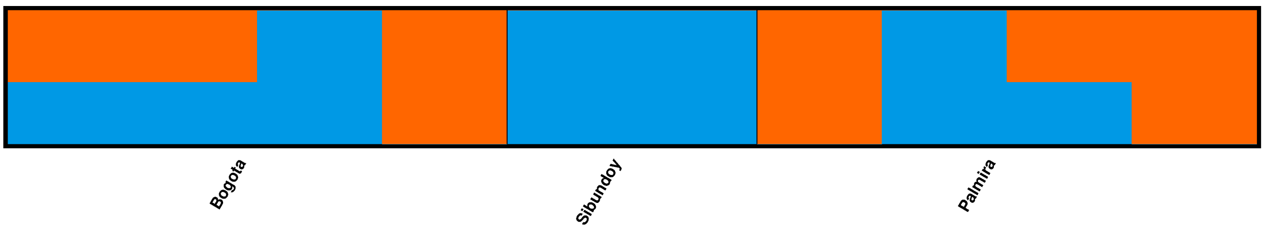

K=3

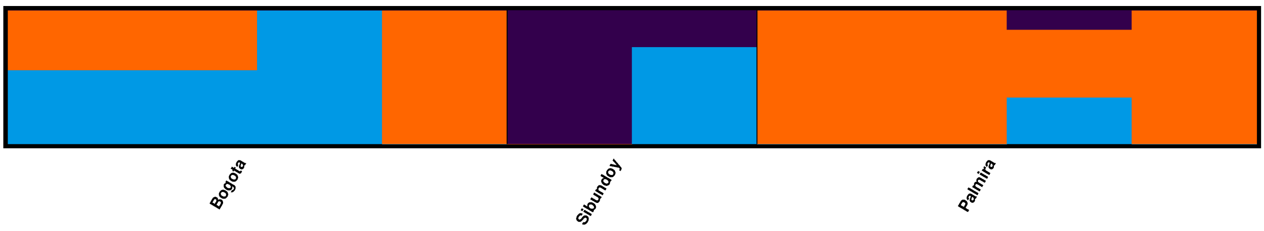

K=4

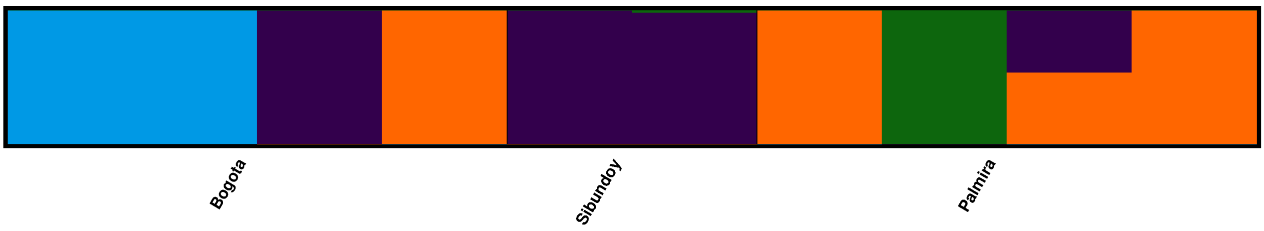

K=5

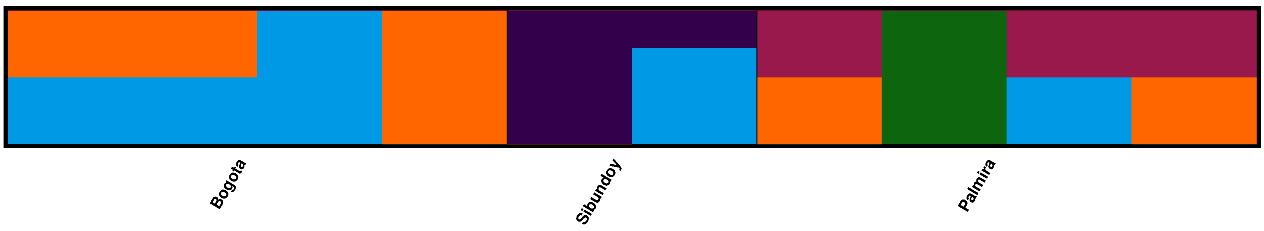

K=6

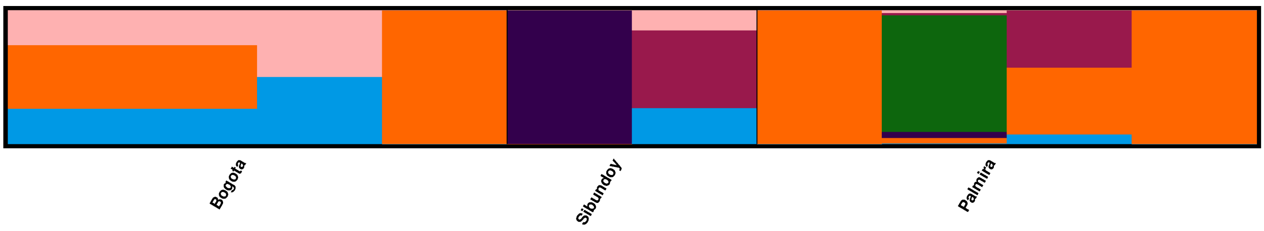

Minor modes for the uploaded data:

Division of runs by mode:

K=1	30/30
K=2	30/30
K=3	30/30
K=4	30/30
K=5	30/30
K=6	30/30
