# Supplementary material for: Exploring Genomics and Microbial Ecology: Analysis of Bidens pilosa L. Genetic Structure and Soil Microbiome Diversity by RAD-Seq and Metabarcoding
Source: Plants (Basel). 2024 Jan 13;13(2):221. doi: 10.3390/plants13020221 (PMC10818919; doi:10.3390/plants13020221)
Supplement: Supplementary file 1 [file plants-13-00221-s001.zip › Figure S3.Venn Diagram.pdf]

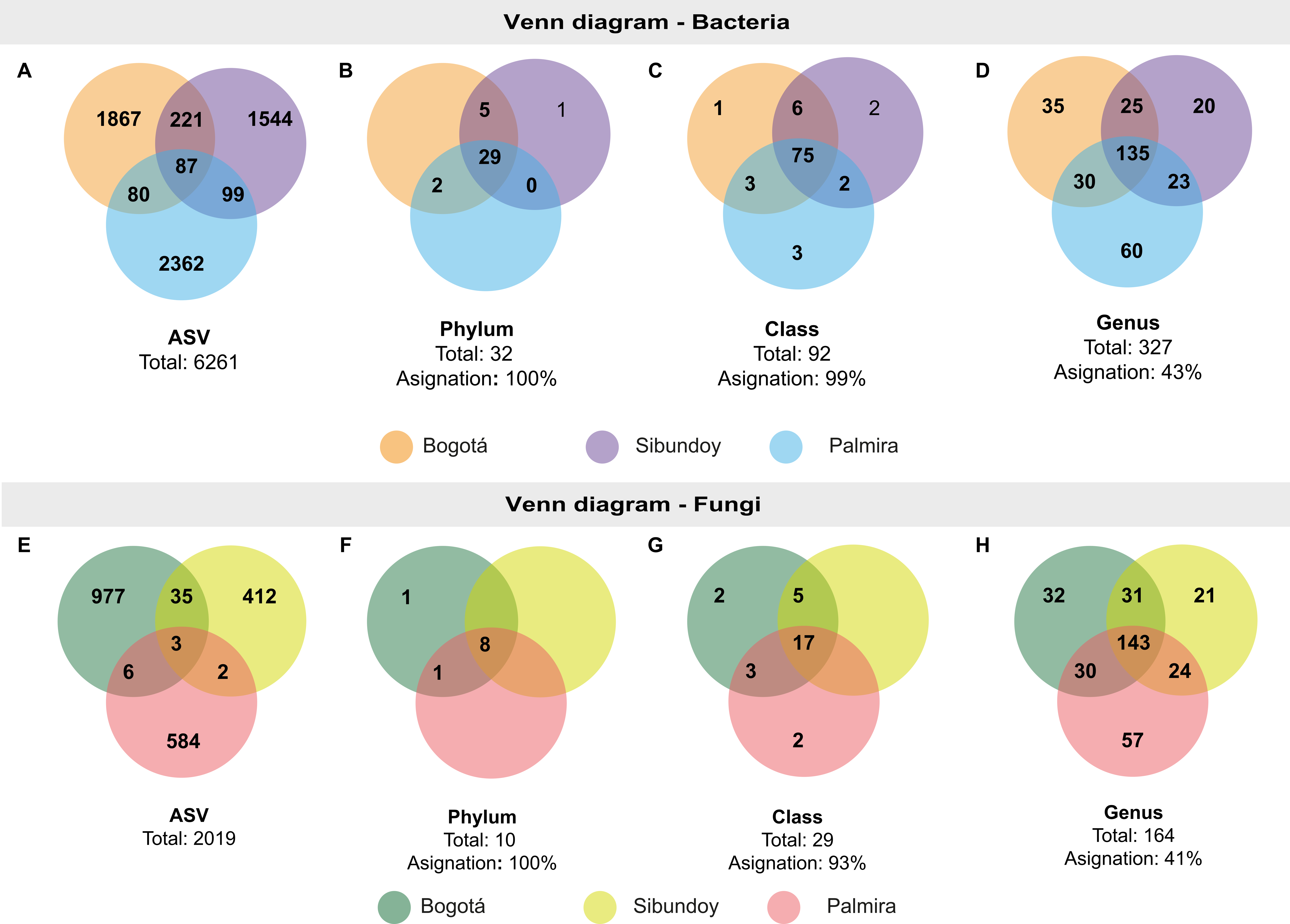

Venn diagram - Bacteria
A
1867
221
1544
87
80
99
2362
ASV
Total: 6261
B
5
1
29
2
0
Phylum
Total: 32
Asignation: 100%
C
1
6
2
75
3
2
3
Class
Total: 92
Asignation: 99%
D
35
25
20
135
30
23
60
Genus
Total: 327
Asignation: 43%
Bogotá
Sibundoy
Palmira
Venn diagram - Fungi
E
977
35
412
3
6
2
584
ASV
Total: 2019
F
1
8
1
Phylum
Total: 10
Asignation: 100%
G
2
5
17
3
2
Class
Total: 29
Asignation: 93%
H
32
31
21
143
30
24
57
Genus
Total: 164
Asignation: 41%
Bogotá
Sibundoy
Palmira
