# Supplementary material for: Exploring Genomics and Microbial Ecology: Analysis of Bidens pilosa L. Genetic Structure and Soil Microbiome Diversity by RAD-Seq and Metabarcoding
Source: Plants (Basel). 2024 Jan 13;13(2):221. doi: 10.3390/plants13020221 (PMC10818919; doi:10.3390/plants13020221)
Supplement: Supplementary file 1 [file plants-13-00221-s001.zip › Table S1. Pipeline Stacks Results.pdf]

**Table S1.** Pipeline Stacks Results

| Barcode | Filename | Fastqc | | | Process_radtags | | | Tsv2bam | gstacks effective_coverages_per_sample | | | |
|---|---|---|---|---|---|---|---|---|---|---|---|---|
| | | Total sequence | Sequence length | %GC | Total RAD | Low Quality | Retained Reads | sample loci to catalog loci | n_loci | n_used_fw_reads | mean_cov | mean_cov_ns |
| GCAATATACATGCAG | cor_1 | 28.784 | 117 | 54 | 2.878.859 | 230 | 2.878.629 | 233.846 | 233.579 | 2.400.847 | 10.279 | 11.671 |
| AAGAATTCGGTGCAG | cor_2 | 18.399 | 117 | 59 | 1.966.710 | 142 | 1.966.568 | 177.946 | 15.419 | 98.047 | 6.359 | 6.427 |
| TCGGCAGTCGTGCAG | cor_3 | 15.499 | 117 | 50 | 1.528.291 | 123 | 1.528.168 | 147.894 | 243.357 | 1.888.196 | 7.759 | 8.312 |
| AGTTCCATTGTGCAG | corsib_1 | 59.528 | 117 | 53 | 4.426.504 | 370 | 4.426.134 | 391.320 | 60.923 | 425.681 | 6.987 | 6.838 |
| TTCTTGCGCTTGCAG | corsib_2 | 8.995 | 117 | 51 | 487.158 | 50 | 487.108 | 58.964 | 656.060 | 6.967.417 | 10.620 | 13.184 |
| AGCAATCTAATGCAG | corsib_3 | 20.077 | 117 | 48 | 1.436.769 | 121 | 1.436.648 | 149.444 | 185.500 | 1.666.463 | 8.984 | 10.116 |
| GAATTGTCGCTGCAG | corsib_4 | 2.565 | 117 | 49 | 187.174 | 20 | 187.154 | 23.805 | 836.628 | 10.159.803 | 12.144 | 16.230 |
| CTTCGACATATGCAG | corsib_5 | 5.086 | 117 | 45 | 379.166 | 28 | 379.138 | 49.265 | 167.002 | 1.113.703 | 6.669 | 7.153 |
| GAGATATGGTTGCAG | sib_1 | 60.973 | 117 | 52 | 3.072.204 | 230 | 3.071.974 | 15.467 | 177.802 | 1.714.945 | 9.645 | 10.186 |
| CTCCTTGGAGTGCAG | sib_2 | 2.760 | 117 | 48 | 123.302 | 10 | 123.292 | 243.825 | 147.689 | 1.288.288 | 8.723 | 9.901 |
| GTGTCTCTTGTGCAG | sib_3 | 33.497 | 117 | 52 | 2.366.944 | 167 | 2.366.777 | 310.503 | 390.768 | 3.784.425 | 9.685 | 10.188 |
| TGCAGTTATCTGCAG | pal_1 | 6.821 | 117 | 53 | 531.273 | 34 | 531.239 | 61.028 | 58.864 | 395.432 | 6.718 | 6.763 |
| TTCTGGAATATGCAG | pal_2 | 125.833 | 117 | 55 | 10.714.124 | 877 | 10.713.247 | 657.154 | 149.235 | 1.152.516 | 7.723 | 8.985 |
| ACGCAACACATGCAG | pal_3 | 19.015 | 117 | 51 | 1.945.096 | 138 | 1.944.958 | 185.713 | 23.740 | 146.828 | 6.185 | 6.323 |
| ACTGCCTCAATGCAG | pal_4 | 215.429 | 117 | 53 | 14.414.533 | 1.057 | 14.413.476 | 838.144 | 49.036 | 294.042 | 5.996 | 6.144 |
| ACATCAATATTGCAG | pal_5 | 18.690 | 117 | 50 | 1.404.006 | 106 | 1.403.900 | 167.378 | 310.155 | 2.649.635 | 8.543 | 9.166 |
