# Supplementary material for: Exploring Genomics and Microbial Ecology: Analysis of Bidens pilosa L. Genetic Structure and Soil Microbiome Diversity by RAD-Seq and Metabarcoding
Source: Plants (Basel). 2024 Jan 13;13(2):221. doi: 10.3390/plants13020221 (PMC10818919; doi:10.3390/plants13020221)
Supplement: Supplementary file 1 [file plants-13-00221-s001.zip › Table S2.K values for samples of three populations.pdf]

Table S2. K values for samples of three populations

Sample	K1	K2	K3
cor_1	0%	45%	55%
cor_3	0%	45%	55%
cor_4	0%	0%	100%
cor_5	0%	100%	0%
sib_1	100%	0%	0%
sib_3	27%	0%	73%
pal_2	0%	100%	0%
pal_3	0%	100%	0%
pal_4	15%	51%	35%
pal_5	0%	100%	0%
