# Supplementary material for: Exploring Genomics and Microbial Ecology: Analysis of Bidens pilosa L. Genetic Structure and Soil Microbiome Diversity by RAD-Seq and Metabarcoding
Source: Plants (Basel). 2024 Jan 13;13(2):221. doi: 10.3390/plants13020221 (PMC10818919; doi:10.3390/plants13020221)
Supplement: Supplementary file 1 [file plants-13-00221-s001.zip › Table S4. Sampling site information.pdf]

Table S4. Sampling site information

Specie	Populations	ID	N individuals	Latitude	Longitude	Altitude (masl)
Bidens pilosa	Juan N Corpas - Bogotá	Cor_1	3	4°45'44.5"	74°05'38.6"	2551
		Cor_2		4°45'44.3"	74°05'38.4"	2551
		Cor_3		4°45'44.1"	74°05'38.0"	2551
		Cor_4	5	4°45'44.5"	74°05'38.6"	2551
		Cor_5		4°45'44.3"	74°05'38.4"	2551
		Cor_6		4°45'44.1"	74°05'38.0"	2551
		Cor_7		4°45'44.1"	74°05'38.0"	2551
		Cor_8		4°45'44.1"	74°05'38.0"	2551
	Sibundoy-Putumayo	Sib_1	3	1° 11' 53.6"	76° 56' 35.6"	2067
		Sib_2		1° 11' 53 .5"	76° 56' 35.6"	2067
		Sib_3		1° 11' 53 4"	76° 56' 35.5"	2067
	Palmira-Valle del Cauca	Pal_1	5	3°25´33.82"	76°25'50.4"	988
		Pal_2		3°25´33.4"	76°25'48.5"	988
		Pal_3		3°25´32.6"	76°25'50.4"	988
		Pal_4		3°25´32.7"	76°25'50.2"	979
		Pal_5		3°25´35.5"	76°25'51.6"	979
