# Supplementary figures and images for: Exploring Genomics and Microbial Ecology: Analysis of Bidens pilosa L. Genetic Structure and Soil Microbiome Diversity by RAD-Seq and Metabarcoding
Source: Plants (Basel). 2024 Jan 13;13(2):221. doi: 10.3390/plants13020221 (PMC10818919; doi:10.3390/plants13020221)

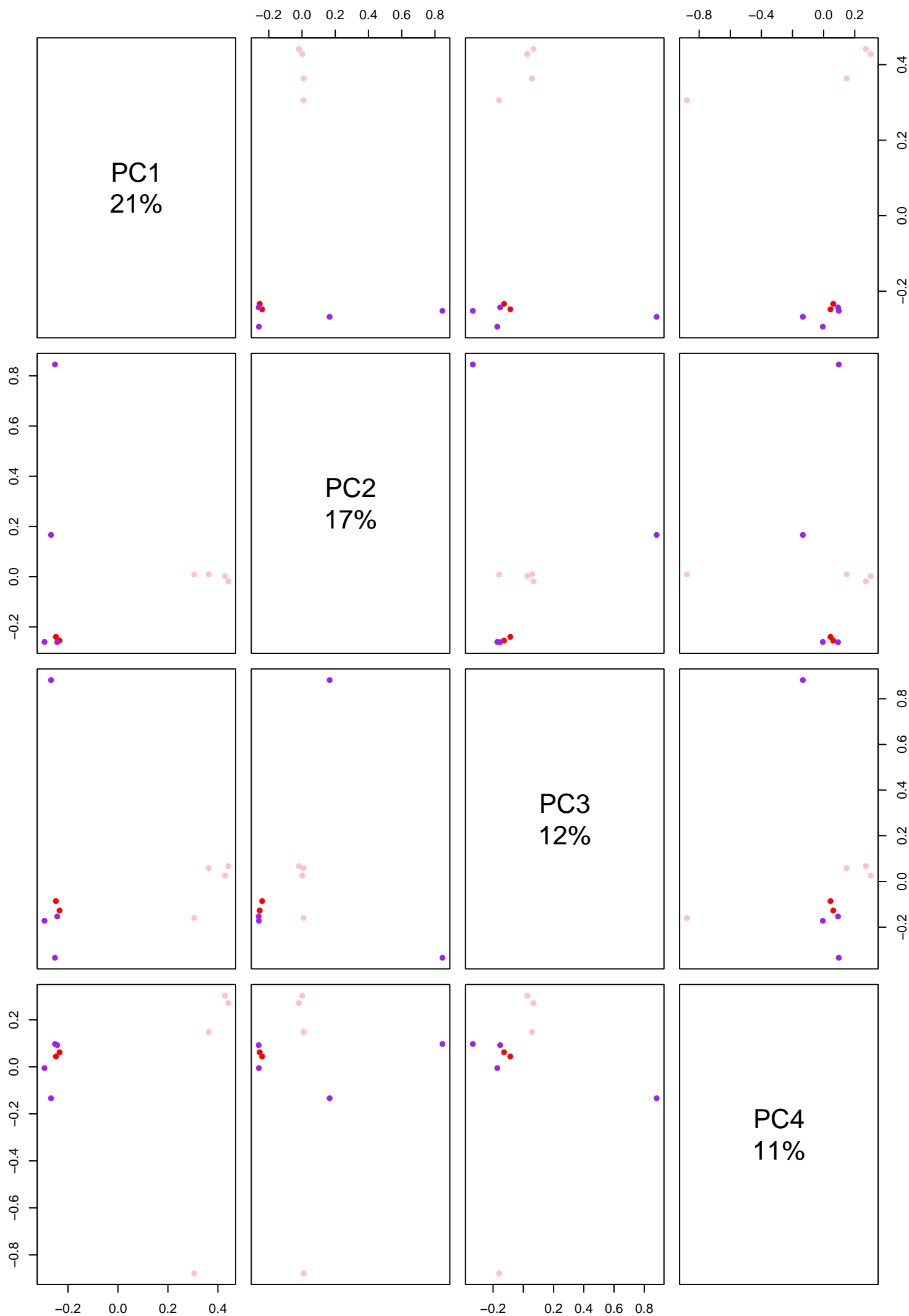

Supplement: Supplementary file 1 [file plants-13-00221-s001.zip › Figure S2. PCA _4C.pdf]

**A****Genus**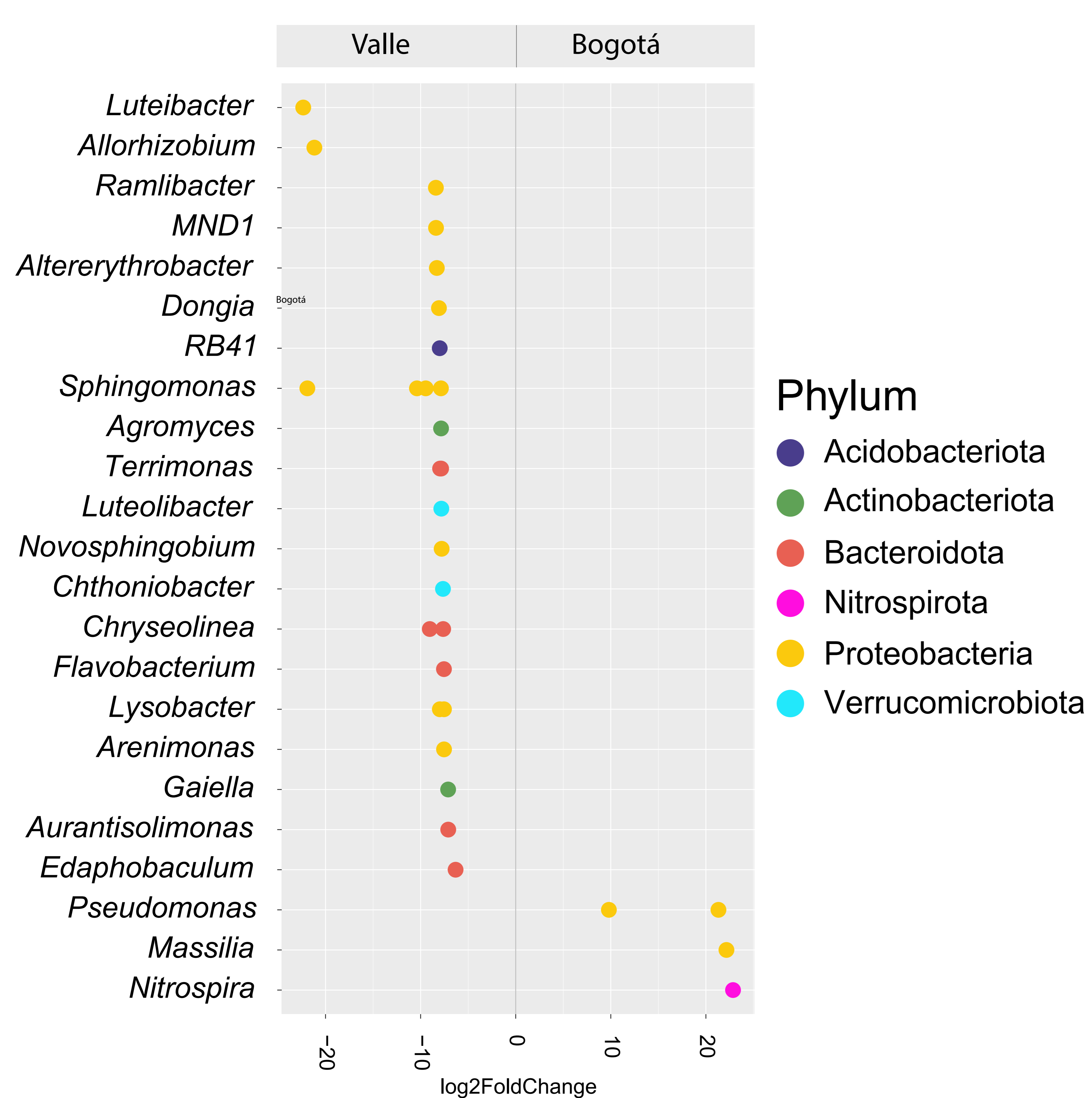**B****Genus**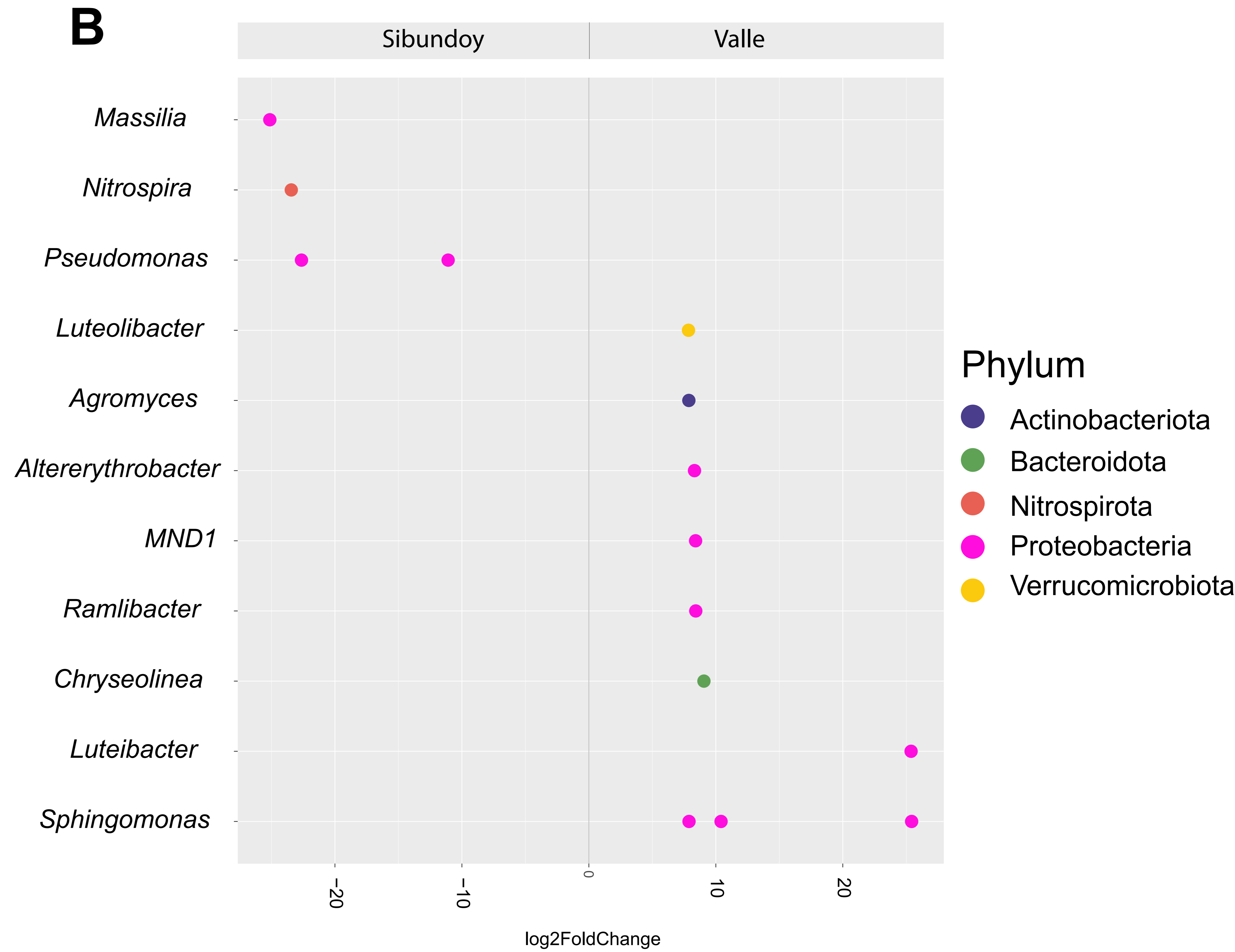**C****Genus**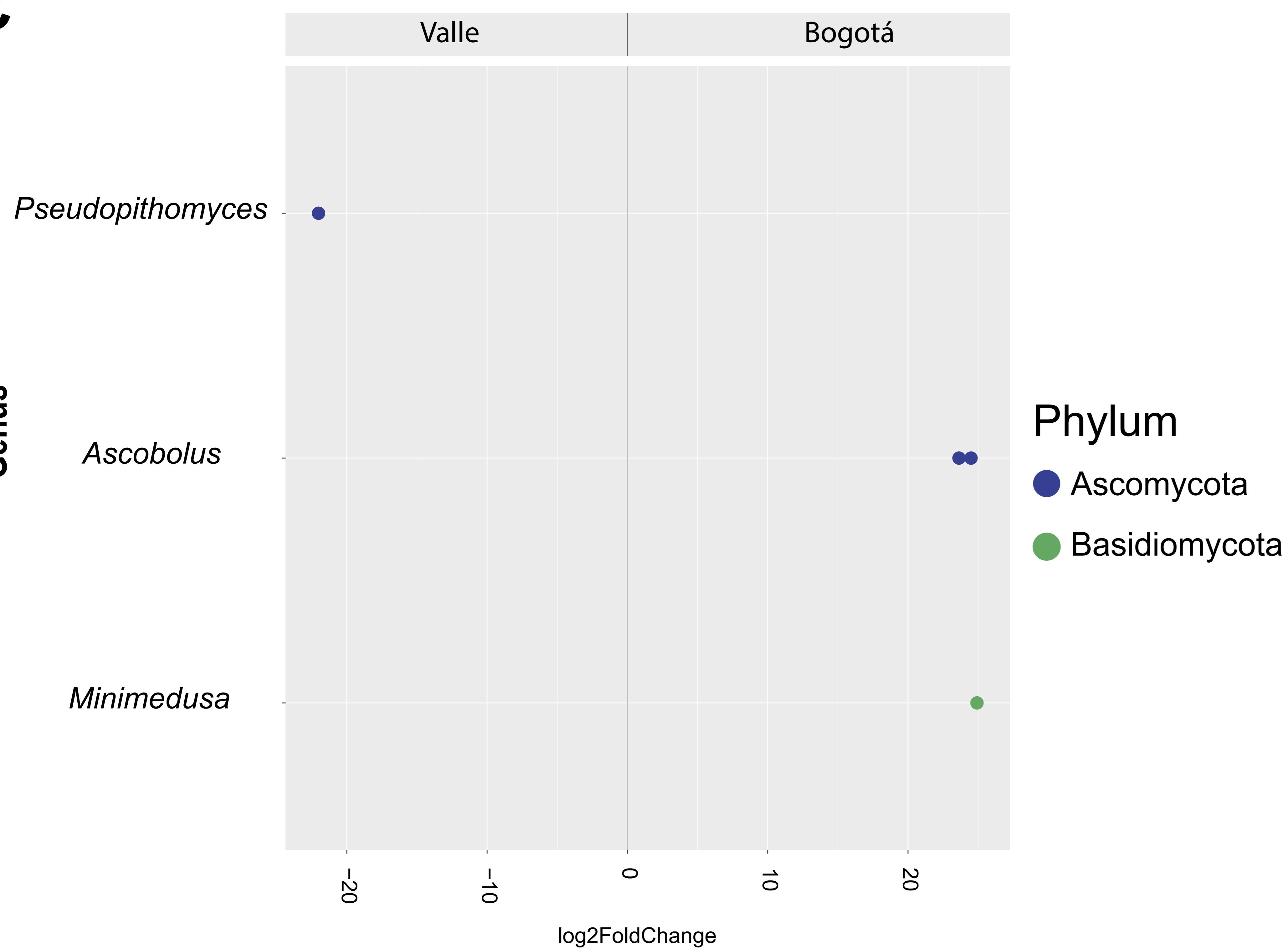**D****Genus**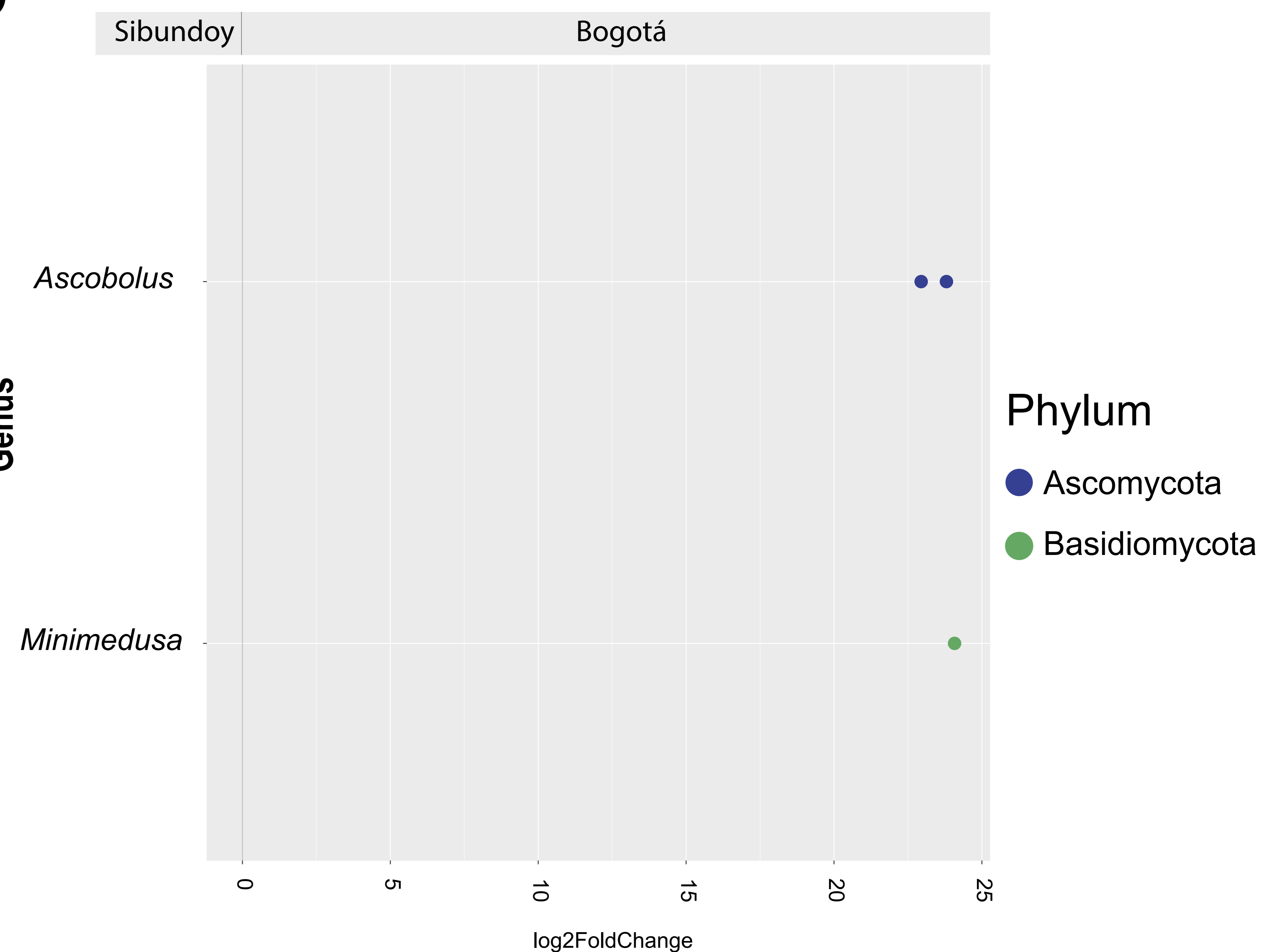

Supplement: Supplementary file 1 [file plants-13-00221-s001.zip › Figure S4. Log2-transformed.pdf]
